# Papillomavirus E6 Oncoproteins Take Common Structural Approaches to Solve Different Biological Problems

**DOI:** 10.1371/journal.ppat.1005138

**Published:** 2015-10-15

**Authors:** Scott Vande Pol

**Affiliations:** Department of Pathology, University of Virginia, Charlottesville, Virginia, United States of America; University of Florida, UNITED STATES

## Introduction

Papillomaviruses induce benign and malignant epithelial tumors in vertebrates through the action of virally encoded oncoproteins such as E6. Recent studies of human and animal E6 proteins determined the three-dimensional structure of E6 when bound to LXXLL peptides derived from E6-targeted cellular proteins. The interaction between diverse E6 proteins and their different cellular targets has revealed a commonality that is surprising and puzzling. E6 proteins that are divergent in evolution have conserved overall structures that bind to similar cellular peptides; these interactions target strikingly different cellular pathways to facilitate analogous viral replication life cycles.

## What Are Papillomaviruses?

Papillomaviruses are small, encapsidated, double-stranded DNA viruses that infect the squamous epithelia of diverse vertebrates, from birds to mammals, and induce neoplasms called papillomas (warts) in which the virus replicates [1]. Papillomas can be visible or inapparent and cutaneous or mucosal. Papillomaviruses are currently grouped into 37 genera, of which five infect humans [2]. A subset of human alpha genus papillomaviruses (alpha HPVs) induce respiratory and anogenital papillomas that can evolve into cancers; they are referred to as “high-risk” HPV types, and the related alpha HPVs that cause stable benign papillomas are called “low-risk” types. Beyond the alpha HPVs, molecular sampling of DNA has revealed a further, vast diversity of HPV types that are typically not associated with visible warts in immune-competent people (beta and gamma genera HPVs) [3]. We live our lives blissfully unaware that we are covered with numerous inapparent warts; we admire ourselves in the mirror, shed the virus into the environment together with our desquamated skin cells, then busily vacuum and sweep the virus into bags for disposal.

## Why Do Papillomaviruses Have Oncogenes?

Papillomaviruses infect squamous epithelial basal cells, where they stably establish the viral genome as a low-copy-number plasmid but allow the infected squamous cells to differentiate, similar to normal stratified squamous epithelia. Productive viral genome amplification occurs in suprabasal cells through DNA damage responses [4], with further differentiation of those cells causing viral capsid protein production and packaging of the infectious virus that is shed into the environment in desquamated cells. This life cycle requires the expression of the early replication genes and the virally encoded oncogenes E5, E6, and E7. Although the alpha genus HPVs make all three oncoproteins, some papillomavirus genera do not: E5 is most often missing, and several papillomavirus types do not make either E6 or E7. Both high-risk HPV E6 and E7 are required for immortalizing primary keratinocytes, maintaining the viral plasmid in infected primary keratinocytes, and maintaining the proliferation of cervical cancer cell lines [3,5]. High-risk alpha E7 represses the retinoblastoma family of tumor suppressors: RB1 (retinoblastoma), RBL1 (p107), and RBL2 (p130); E7 targets the degradation of RB1, which is tied to the cancer phenotype [5]. High-risk HPV E7 degradation of RB1 stabilizes p53, which is then degraded by high-risk E6 [6].

## What Cellular Proteins Associate with Alpha Genus E6?

When high-risk E7 targets the degradation of RB1, the p53 tumor suppressor is stabilized but then degraded by high-risk E6 through its association with a cellular E3 ubiquitin ligase called E6-Associated Protein, or E6AP (product of UBE3A gene); neither E6 nor E6AP alone associate with p53, but p53 is recruited to the E6+E6AP complex, ubiquitinated, and then degraded by the proteasome [7]. E6 binds to E6AP by docking on a ten-amino-acid acidic alpha-helical peptide referred to as LXXLL [7]; the binding of E6 to the LXXLL peptide of E6AP alone is sufficient to stabilize E6 in vivo and change the structure of E6 so it can then associate with the secondary substrate, p53 [8]. High-risk alpha HPV E6 also displays a peptide at the C-terminus of E6 that interacts with a set of cellular PDZ proteins that includes DLG family proteins, scribble, nonreceptor tyrosine phosphatases and others; the E6-associated PDZ proteins can then be subject to ubiquitination by E6AP and degradation by the proteasome, although the requirement for degradation by the proteasome is unclear [9]. Although low-risk alpha E6 proteins also bind to E6AP, those E6 proteins neither associate with p53 nor bind cellular PDZ proteins [3]. But, like high-risk alpha E6, low-risk E6 is also required to maintain the viral genome as a plasmid [10]. High-risk alpha E6 proteins have additional important biological effects, such as modulation of RNA transcription and translation, metabolism, apoptosis, and autophagy that are beyond the scope of this micro-review [3]. An unbiased proteomic analysis of cellular proteins that associate with the alpha and beta E6 proteins has identified additional, as yet uncharacterized E6-associated proteins [11].

## What Cellular Proteins Associate with Non-alpha Genera E6 Proteins?

Recent work in three labs identified MAML family transcriptional coactivators as targets of beta and mu genera HPV and delta genus bovine papillomavirus type 1 (BPV1) E6 proteins [12–14]. Like alpha E6 proteins that dock on an acidic LXXLL peptide of E6AP, the delta/beta/mu genera E6 proteins also bind an acidic LXXLL peptide found at the C-terminus of MAML family coactivators, thereby repressing the transcriptional activation function of MAML [12,14]. MAML proteins are the transcriptional coactivators for Notch signal transduction; Notch signaling drives the differentiation of squamous epithelia, and Notch signaling is a tumor suppressor in squamous epithelia [15]. E6 association with MAML thus represses Notch signaling and thereby represses squamous differentiation. Interestingly, in people with mutation of their EVER1 or EVER2 genes, beta genus HPV-caused warts are visibly apparent and can progress to squamous cell cancers in sun-exposed areas; this reveals the potential of beta genus HPV to contribute to carcinogenesis [16]. Along this line, beta genus E6 proteins block normal excision repair of UV thymidine dimers [17]. Some beta genus HPV E6 also associate with p300/CBP and the Ccr4-Not complex, but whether these are primary LXXLL-like interactions or secondary interactions is currently unknown [11]. In addition to MAML, the delta genus BPV1 E6 also associates with the signaling adaptor protein paxillin by binding to acidic LXXLL peptides in paxillin; expression of paxillin is required for BPV1 E6 transformation [18].

## What Is the Structure of E6, and What Do Different E6 Proteins Have in Common?

**Fig 1:**
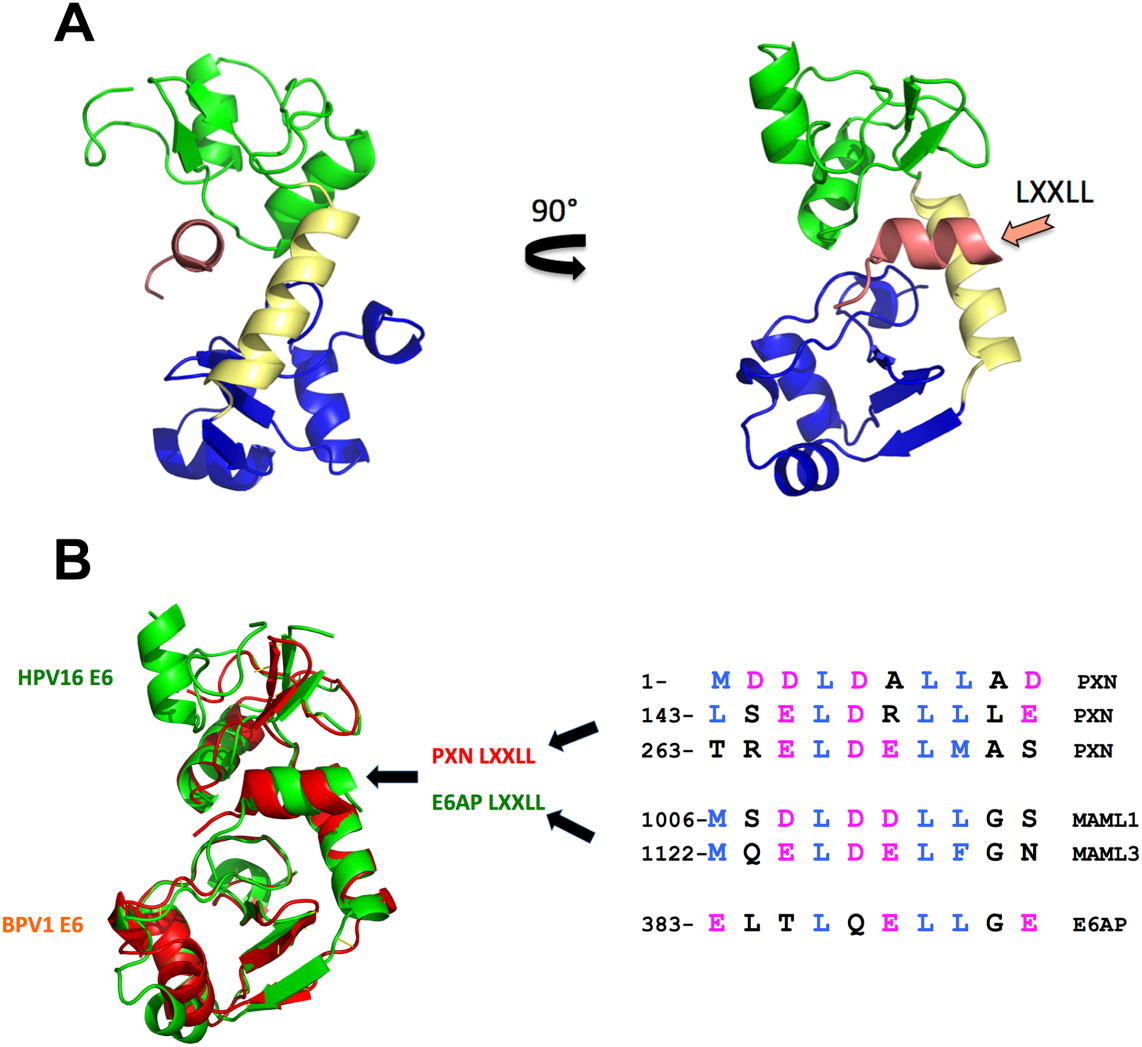
E6 structure and LXXLL interactions. A. Ribbon diagram of HPV16 E6 bound to the LXXLL motif from E6AP [20]. The amino-terminal zinc-binding domain (16E6-N in green) is connected by an alpha helix (yellow) to the E6-C zinc-binding domain at the bottom (blue). The helical LXXLL peptide from the cellular E6AP ubiquitin ligase (salmon color) resides in a deep 16E6 pocket. B. Divergent E6 proteins have conserved folds. Despite the limited homology (30% identity), the evolutionary divergence of human and bovine hosts, and different interacting cellular protein targets, superimposition of HPV16 E6 (green) and BPV1 E6 (red) structures demonstrates the conservation of the overall fold of E6 and its interaction with the cellular protein–derived LXXLL peptides. The paxillin (PXN) and E6AP-LXXLL peptides in the structure are colored with their respective E6 protein, and known interacting LXXLL peptide sequences are shown to the right (hydrophobic side chains in blue and acidic side chains in purple).

All E6 proteins examined so far bind cellular proteins by docking on similar acidic LXXLL peptides (Fig 1B), even if those targets (E6AP, MAML, and paxillin) are seemingly unrelated. The E6–LXXLL peptide interaction is necessary for alpha E6 proteins to be stable in cells and to bind and degrade p53 [7]; BPV1 E6 and beta genera HPV E6 must bind LXXLL to repress Notch signaling [12,14], and this interaction is necessary for BPV1 E6 to transform cells [19]. E6 alone is insoluble when expressed in bacteria, which precluded structural studies for decades, but the E6–LXXLL complex is soluble, allowing these structures to be determined by the Trave lab [20]. The precise structure of E6 unbound to LXXLL remains undetermined. In each structure, the LXXLL peptide is in a deep E6 pocket and contacts the amino terminus (green), carboxy terminus (blue), and connecting helix (yellow) of E6 (Fig 1A). Despite the divergent primary sequence between BPV1 and HPV16 E6 (only 30% sequence identity), the overall fold is strikingly similar (Fig 1B); thus, the hundreds of human and animal papillomavirus E6 proteins whose cellular targets are as yet unknown will also interact with acidic amphipathic helical peptides analogous to the LXXLL motifs of MAML, E6AP, and paxillin (Fig 1B) [20].

## Summary, and What Is Still Unknown?

### For all E6 proteins

1. Structure and sequence analysis allows us to predict that most E6 proteins will interact with similar acidic alpha-helical peptides. The E6 proteins studied so far have a conserved fold that binds an acidic LXXLL peptide on either E6AP, (alpha genus), MAML (beta and delta genera,) or paxillin (delta genus). Do the other genera of E6 proteins have these same three LXXLL targets, or is there a vast array of cellular proteins with acidic LXXLL docking sites yet to be discovered? Is there some underlying biological logic to the association of E6 with acidic LXXLL motifs?
2. What, in an evolutionary context, made a particular genus of E6 protein “choose” either E6AP or MAML as a target? Is there a distinct difference in the biology of particular squamous epithelial niches, such as cutaneous versus mucosal, follicular versus interfollicular cells, or squamous–columnar junctions?
3. Small molecules or inhibitory peptides that compete for binding to the LXXLL-binding pocket of E6 block E6 function in vitro and in vivo. Could potent inhibitors of the LXXLL pocket be therapeutic for papillomavirus infections and papillomavirus-caused malignancies?

### Alpha genus HPV E6 proteins

1. When high-risk E6 binds LXXLL, E6 is stabilized and solubilized, and restructures to interact with p53. Do E6 proteins from low-risk alpha (or other genera) also have secondary substrates that bind after the E6–LXXLL interaction? Do low-risk alpha E6 proteins target the degradation of cellular proteins?
2. Is it possible there might be a biological role for E6+E6AP complexes that do not trigger ubiquitination and protein degradation?
3. High-risk alpha E6 proteins have a set of at least three activities that are not found in other E6 types: p53 degradation, PDZ protein association, and telomerase induction. Are all three functions necessary for virus replication or cancer formation by E6 + E7, and how are they linked to each other and to activities of E7? Low-risk alpha and other studied papillomavirus genera E6 proteins do not induce telomerase, and yet these other viruses all make fine warts. Why does only high-risk E6 induce telomerase?
4. High-risk alpha E6 proteins interact with a set of cellular PDZ proteins. Which PDZ target(s) is required for the viral life cycle? Is degradation of the PDZ target by E6 important?
5. What structural features of alpha genus E6 contribute to the evasion of effective immunity by the host?
6. E6 proteins are required to maintain the viral genome in keratinocytes in both high and low-risk alpha genus. For the high-risk E6, p53 degradation and PDZ association are required for this, but are there other activities in common with the low-risk E6 proteins, and what are the low-risk E6 proteins doing?

### For beta and delta genera E6 proteins

1. How does the repression of Notch signaling by E6 cooperate with the activities of E7 to promote virus replication?
2. When E6 proteins bind LXXLL on MAML, do they recruit secondary substrates to the E6+MAML complex, as the high-risk E6 proteins do when they bind E6AP?
3. Since Notch signaling is required for squamous epithelial differentiation, how is the E6 repression of Notch signaling negatively modulated to allow for epithelial differentiation and capsid protein expression? Notch is a tumor suppressor in skin, and disruption of Notch signaling in mice causes squamous cell cancers. Do papillomavirus types with E6 genes that repress Notch contribute to the development of squamous cell cancers in normal people?

Recent studies have elucidated much that was once unfocused about E6, but the above questions indicate we should look closer still, and put on some better glasses.

## References

[1] Rector A, Van Ranst M (2013) Animal papillomaviruses. Virology 445: 213–223. 10.1016/j.virol.2013.05.007 23711385

[2] Van Doorslaer K (2013) Evolution of the papillomaviridae. Virology 445: 11–20. 10.1016/j.virol.2013.05.012 23769415

[3] Vande Pol SB, Klingelhutz AJ (2013) Papillomavirus E6 oncoproteins. Virology 445: 115–137. 10.1016/j.virol.2013.04.026 23711382 PMC3783570

[4] Moody CA, Laimins LA (2009) Human papillomaviruses activate the ATM DNA damage pathway for viral genome amplification upon differentiation. PLoS Pathog 5: e1000605 10.1371/journal.ppat.1000605 19798429 PMC2745661

[5] Roman A, Munger K (2013) The papillomavirus E7 proteins. Virology 445: 138–168. 10.1016/j.virol.2013.04.013 23731972 PMC3783579

[6] Scheffner M, Werness BA, Huibregtse JM, Levine AJ, Howley PM (1990) The E6 oncoprotein encoded by human papillomavirus types 16 and 18 promotes the degradation of p53. Cell 63: 1129–1136. 2175676 10.1016/0092-8674(90)90409-8

[7] Huibregtse JM, Scheffner M, Howley PM (1993) Localization of the E6-AP regions that direct human papillomavirus E6 binding, association with p53, and ubiquitination of associated proteins. Mol Cell Biol 13: 4918–4927. 8393140 10.1128/mcb.13.8.4918-4927.1993 PMC360130

[8] Ansari T, Brimer N, Vande Pol SB (2012) Peptide interactions stabilize and restructure human papillomavirus type 16 E6 to interact with p53. J Virol 86: 11386–11391. 22896608 10.1128/JVI.01236-12 PMC3457172

[9] Pim D, Bergant M, Boon SS, Ganti K, Kranjec C, et al (2012) Human papillomaviruses and the specificity of PDZ domain targeting. FEBS J 279: 3530–3537. 10.1111/j.1742-4658.2012.08709.x 22805590

[10] Oh ST, Longworth MS, Laimins LA (2004) Roles of the E6 and E7 proteins in the life cycle of low-risk human papillomavirus type 11. J Virol 78: 2620–2626. 14963169 10.1128/JVI.78.5.2620-2626.2004 PMC369251

[11] White EA, Kramer RE, Tan MJ, Hayes SD, Harper JW, et al (2012) Comprehensive analysis of host cellular interactions with human papillomavirus E6 proteins identifies new E6 binding partners and reflects viral diversity. J Virol 86: 13174–13186. 10.1128/JVI.02172-12 23015706 PMC3503137

[12] Brimer N, Lyons C, Wallberg AE, Vande Pol SB (2012) Cutaneous papillomavirus E6 oncoproteins associate with MAML1 to repress transactivation and NOTCH signaling. Oncogene 31: 4639–4646. 10.1038/onc.2011.589 22249263 PMC3330202

[13] Rozenblatt-Rosen O, Deo RC, Padi M, Adelmant G, Calderwood MA, et al (2012) Interpreting cancer genomes using systematic host network perturbations by tumour virus proteins. Nature 487: 491–495. 10.1038/nature11288 22810586 PMC3408847

[14] Tan MJ, White EA, Sowa ME, Harper JW, Aster JC, et al (2012) Cutaneous beta-human papillomavirus E6 proteins bind Mastermind-like coactivators and repress Notch signaling. Proc Natl Acad Sci U S A 109: E1473–1480. 10.1073/pnas.1205991109 22547818 PMC3384212

[15] Nicolas M, Wolfer A, Raj K, Kummer JA, Mill P, et al (2003) Notch1 functions as a tumor suppressor in mouse skin. Nat Genet 33: 416–421. 12590261 10.1038/ng1099

[16] Ramoz N, Rueda LA, Bouadjar B, Montoya LS, Orth G, et al (2002) Mutations in two adjacent novel genes are associated with epidermodysplasia verruciformis. Nat Genet 32: 579–581. 12426567 10.1038/ng1044

[17] Wallace NA, Robinson K, Howie HL, Galloway DA (2012) HPV 5 and 8 E6 abrogate ATR activity resulting in increased persistence of UVB induced DNA damage. PLoS Pathog 8: e1002807 10.1371/journal.ppat.1002807 22807682 PMC3395675

[18] Wade R, Brimer N, Vande Pol S (2008) Transformation by bovine papillomavirus type 1 E6 requires paxillin. J Virol 82: 5962–5966. 10.1128/JVI.02747-07 18385245 PMC2395150

[19] Bohl J, Das K, Dasgupta B, Vande Pol SB (2000) Competitive binding to a charged leucine motif represses transformation by a papillomavirus E6 oncoprotein. Virology 271: 163–170. 10814581 10.1006/viro.2000.0316

[20] Zanier K, Charbonnier S, Sidi AO, McEwen AG, Ferrario MG, et al (2013) Structural basis for hijacking of cellular LxxLL motifs by papillomavirus E6 oncoproteins. Science 339: 694–698. 10.1126/science.1229934 23393263 PMC3899395

